# A comparison between the APACHE II and Charlson Index Score for predicting hospital mortality in critically ill patients

**DOI:** 10.1186/1472-6963-9-129

**Published:** 2009-07-30

**Authors:** Susan Quach, Deirdre A Hennessy, Peter Faris, Andrew Fong, Hude Quan, Christopher Doig

**Affiliations:** 1 Department of Community Health Sciences, University of Calgary, Calgary, Alberta, Canada; 2 Department of Critical Care Medicine, University of Calgary, Calgary, Alberta, Canada

## Abstract

**Background:**

Risk adjustment and mortality prediction in studies of critical care are usually performed using acuity of illness scores, such as Acute Physiology and Chronic Health Evaluation II (APACHE II), which emphasize physiological derangement. Common risk adjustment systems used in administrative datasets, like the Charlson index, are entirely based on the presence of co-morbid illnesses. The purpose of this study was to compare the discriminative ability of the Charlson index to the APACHE II in predicting hospital mortality in adult multisystem ICU patients.

**Methods:**

This was a population-based cohort design. The study sample consisted of adult (>17 years of age) residents of the Calgary Health Region admitted to a multisystem ICU between April 2002 and March 2004. Clinical data were collected prospectively and linked to hospital outcome data. Multiple regression analyses were used to compare the performance of APACHE II and the Charlson index.

**Results:**

The Charlson index was a poor predictor of mortality (C = 0.626). There was minimal difference between a baseline model containing age, sex and acute physiology score (C = 0.74) and models containing either chronic health points (C = 0.76) or Charlson index variations (C = 0.75, 0.76, 0.77). No important improvement in prediction occurred when the Charlson index was added to the full APACHE II model (C = 0.808 to C = 0.813).

**Conclusion:**

The Charlson index does not perform as well as the APACHE II in predicting hospital mortality in ICU patients. However, when acuity of illness scores are unavailable or are not recorded in a standard way, the Charlson index might be considered as an alternative method of risk adjustment and therefore facilitate comparisons between intensive care units.

## Background

In health services research, risk adjustment is important to account for variations in case-mix that might affect assessment of outcomes, therefore allowing comparisons to be made between health care providers and health systems locally and globally. In the intensive care unit (ICU), risk adjustment and mortality prediction has usually been performed using severity score taxonomies such as the Acute Physiology and Chronic Health Evaluation (APACHE) score, the Simplified Acute Physiology Score (SAPS) or the Mortality Prediction Model (MPM) [1-3], and their updated derivatives [4-8]. These systems emphasize the severity of physiologic derangement, but also include, to a variable degree and weight, some measure of pre-existing illness. For example, the APACHE II system assigns 0, 2 or 5 chronic health points (CHP) for pre-existing co-morbidity out of a possible 73 total points [4]. Since the emphasis of all these systems is the measurement of physiological derangement, the small consideration of pre-existing disease may not adequately account for the quantitative or qualitative contribution of co-morbid illness, thus limiting the ability of these models to accurately predict mortality [9].

A significant limitation to severity of illness scores is that data collection is labour intensive and expensive, and the necessary data to calculate these scores are unlikely to be found in administrative databases. Therefore, the utility of these scores in risk adjustment is limited to prospective studies including clinical trials. It is unlikely that these scores can be accurately calculated for use in retrospective studies such as for benchmarking, quality assurance, or to examine differences in service delivery within a city, country or between countries. Other systems, used widely outside critical care, have been developed to predict patient outcomes based solely on the presence of predefined existing co-morbidities. For instance the Charlson index assigns weighted scores to 17 co-morbidities ranging from chronic heart failure to HIV infection [10]. Classification within systems like the Charlson index originally required a chart review, and therefore was also costly and labour intensive. However, recent work has validated the determination of these scores from administrative data, permitting quick, easy, and inexpensive calculation. Therefore, these scores can be determined for prospective and retrospective studies.

Small studies have suggested that the Charlson index may perform comparably or better than APACHE II for mortality prediction in an ICU population [11,12]. Poses et al [11] suggested that the Charlson index could improve prognostic predictions in ICU patients when added to the APACHE II. However, their study was conducted in only one hospital with data collected manually by chart review over a short interval. There is little data on the validity and utility of the Charlson index, calculated using administrative data, as a risk adjustment method for ICU patients [13]. Therefore, the purpose of this study was to compare the performance of the Charlson index alone and in combination with components of APACHE II for predicting hospital mortality among adult ICU patients.

## Methods

### Study Population

The Calgary Health Region (CHR) exclusively provides in-hospital acute care to all residents of the Calgary Health Region (city of Calgary and approximately 20 nearby small towns, villages, and hamlets total population approximately 990,000 in 2002). The CHR Department of Critical Care Medicine provides care to critically ill patients in three closed multidisciplinary and 1 closed cardiovascular intensive care units (ICUs). The base study population consisted of all adult (>17 years) CHR residents admitted to any multidisciplinary ICU in the CHR between April 1st 2002 and March 31st 2004. Cardiovascular surgery patients (n = 2541) were excluded because they differed from other ICU patients with respect to their physiologic derangement, mortality rate, and the utility of the APACHE II system for predicting mortality in this subgroup. During the study period, the first admission of a patient to the ICU and their subsequent hospital stay was considered the index admission. All data were analyzed for the index admission only. The study was approved by the local human research ethics committee. Individual patient consent was waived as the final study sample contained no individual patient identifiers following the linkage described below.

### Design and Data Sources

This study used a population-based cohort design that linked clinical and outcome data from two large administrative databases. Demographic, physiological, diagnostic and treatment data for study participants during their ICU stay were recorded prospectively using the Quantitative Sentinel (QS) 6.6.9 Clinical Information System (GE Marquette Medical Systems INC., Milwaukee, WI) and stored in a locally developed longitudinal relational database called ICU Tracer, which is built on an Oracle Enterprise platform (Oracle Corporation, Redwood Shores, CA). Details about the Tracer database have been previously published [14]. Patients were identified in the Tracer database, and their demographics and clinical data were then linked to hospital discharge data obtained from the CHR administrative database through a deterministic record linkage using last name, date of birth, ICU admit date, ICU discharge date and unique provincial healthcare number (PHN). The administrative hospital discharge data contained up to 16 secondary diagnosis codes and types as well as 10 procedure codes, which were used to derive the Charlson index. Using the ICD-10-CM code taxonomy to identify diagnoses and procedures, we excluded the diagnosis type with M (main diagnosis) or a 2 (post admit co-morbidity) because these conditions were the cause or consequence of the hospital admission, and not a pre-existing comorbidity. The linkage rate was 99%. All individual patient personal identifiers were stripped following linkage.

### Main Outcome Measure and Variables

The primary outcome was all cause hospital mortality. The APACHE II score was collected prospectively at the time of ICU admission in accordance with the original published recommendations [4]. At ICU admission, the attending critical care physician, using a standardized automated entry system with on-line operational definitions, determined the diagnosis responsible for ICU admission, the Glasgow coma score (based on the best assessment of neurological status excluding any potential confounding effect of medications), and the CHP component. The physiologic and laboratory data required to calculate the acute physiology score (APS) component of the APACHE II were obtained by automated electronic download from physiologic monitors or laboratory information systems. Variables from physiologic monitors required validation by nursing staff prior to download. The calculation of APS was based on the worst value for each variable within the 24 hours of ICU admission. The Charlson index consists of 17 co-morbidities, which are weighted and summed to produce a score [10]. We used the ICD-10 coding algorithms developed by Quan *et al*. [15] to derive a co-morbidity score for each patient. In this multi-step process, ICD-10 coding algorithms were developed by translating the ICD-9-CM codes derived from Deyo's method [16]. The validity of the algorithms for ICD-10 coding from administrative data has been previously reported [15].

### Statistical Analysis

Baseline characteristics including the frequency of Charlson comorbidities were calculated for the cohort of patients. Univariate analysis was used to compute the C-statistic for models containing CHP, Charlson index score and APACHE II alone. Four separate nested multivariate logistic regression models were constructed. The baseline model consisted of age, sex and APS (Model A). To this baseline model, were then added individually CHP (Model B), the Charlson co-morbidities as individual entities (Model C), and the Charlson index as a weighted score in the same categories as suggested by D'Hoore (1–2, 3–4, 5–6, >6) (Model D) [17]. Finally, as the relationship between the Charlson index and the log odds of death was non-linear the Charlson index was recoded excluding scores of 6 and above (Model E). Finally, the discriminative ability of the full APACHE II score (Model F) was examined with the addition of the weighted Charlson index score (Model G). The C-statistic was used to discriminate between patients who would live and die and represents the area under the curve for the probability that the mortality status of a randomly selected survivor and non survivor is identified correctly by a given prediction model [18]. C-statistics of 0.8 to 0.9 are regarded as very good, 0.7 to 0.8 are considered adequate and C-statistics below 0.7 are regarded as poor [19]. To test whether the addition of co-morbidity variables improved the explanatory ability of the nested models changed significantly, likelihood ratio tests (LR) were used [20]. The LR test was computed as twice the difference between the log-likelihood from the two models being compared, and the statistic derived was compared to a χ^2 ^(chi-squared) distribution for significance testing. Since model performance can be degraded by inclusion of many predictors, we calculated Akaike's Information Criterion (AIC). This statistic provides a method for penalizing the log likelihood to gain an unbiased assessment of the model's performance after inclusion of additional predictors. To calculate the AIC, we added twice the number of parameters to the -2 log likelihood [20]. Goodness of fit was assessed by graphical means and by the Hosmer and Lemeshow's Goodness of Fit test. Finally, Efron's enhanced bootstrapping method was performed to estimate the amount of shrinkage in the models [21]. Finally, we explored the effect of re-weighting the individual co-morbidities within the Charlson index based on their odds ratio of being associated with hospital mortality. All statistical analyses were preformed using SAS version 9.1 (Cary, NC) and STATA 9.2 (College Station, Texas).

## Results

During the two year study period, there were 3778 eligible index admissions into the multi-disciplinary ICUs. Patient characteristics are described in Table 1. Patients had an average age of 56.9 years, with a slightly higher percentage of males (57.0%). The mean (± SD) admitting APACHE II and APS scores were 19.6 (± 8.6) and 15.2 (± 7.7) respectively. The ICU and hospital mortality rates were 18.9% and 27.2% respectively. According to the CHP classification 26.5% of patients had severe chronic health problems. The Charlson index was highly skewed with 77.0% of the observations between 0 and 2. Forty one percent of patients (41.3%) had no co-morbidities, 28.8% had a single co-morbidity, 25.6% had two or three co-morbidities and 3.7% of patients had four or more co-morbidities.

**Table 1:** Patient Characteristics

Age (y)	
Mean ± SD*	56.9 ± 19.7
Sex (%)	
Male	2142 (57.0)
Female	1615 (43.0)
Unknown	21 (0.50)
Length of ICU stay (days)	
Median (IQR)†	2.8 (1.2,6.4)
Admitting APACHE II Score	
Mean ± SD	19.6 ± 8.6
First TISS score‡	
Mean ± SD*	35.0 ± 14.0
In hospital Mortality (%)	
Frequency	1026 (27.2)
ICU mortality (%)	
Frequency	712 (18.9)
Charlson Index Score	
Median (IQR; maximum)†	1 (0,2;13)
Chronic Health Points (%)	
0	2643 (70.0)
2	95 (2.5)
5	985 (26.1)
Unknown	55 (1.5)
APS	
Mean ± SD	15.2 ± 7.7

Total sample size = 3778, unless otherwise stated.

*SD = standard deviation

†IQR = interquartile range

‡TISS = Therapeutic Intervention Scoring System

The prevalence of co-morbidities, the Charlson weight, and the association of the co-morbidity with hospital mortality are presented in Table 2. The most common co-morbidity was either chronic pulmonary or cardiac disease, followed by diabetes. The only disease with a prevalence of less than 1% was known pre-existing seropositive HIV status. In the original Charlson classification, co-morbidities with odds ratio associated with mortality of ≥1.2<1.5 were assigned a weight of 1, conditions with a ratio of ≥1.5<2.5 a weight of 2, conditions with ≥2.5<3.5 a weight of 3, conditions with ≥3.5<4.5 a weight of 4, and those conditions with weights of 6 or more were assigned a weight of 6. A 'recoded' ICU weight based on the odds-ratio of the co-morbidity demonstrated in this study sample that 7 of the co-morbidities would be assigned a weight of zero, 3 co-morbidities (cerebrovascular disease and liver disease (mild and moderate or severe)) would have a larger weight, 4 variables would have smaller weights (cancer and metastatic cancer, HIV seropositive status, and paraplegia-hemiplegia) and only 2 co-morbidities would not change their weight (myocardial infarction and peripheral vascular disease).

**Table 2:** Estimation of logistic regression parameters to predict in-hospital death

**Co-morbidity**	**Charlson weight**	**Persons with co-morbidity(%)**	**OR (95%CI)**	**ICU weight †**	**Hospital mortality associated with individual co-morbidity(%)**
Chronic pulmonary disease	1	16.38	0.84 (0.67, 1.04)		27.46
Congestive heart failure	1	12.92	1.06 (0.84, 1.33)		35.86
Diabetes without complications	1	12.44	0.77 (0.61, 0.98)		29.15
Myocardial infarction	1	10.03	1.47 (1.15, 1.89)	1	40.94
Renal disease	2	7.41	1.37 (1.02, 1.84)	1	40.00
Cerebrovascular disease	1	6.14	1.75 (1.28, 2.38)	2	40.94
Cancer	2	5.64	1.46 (1.06, 2.00)	1	40.38
Peripheral vascular disease	1	5.61	1.30 (0.94, 1.80)	1	41.51
Paraplegia and hemiplegia	2	4.71	1.27 (0.88, 1.83)	1	32.02
Metastatic carcinoma	6	5.64	1.50 (1.06, 2.13)	2	62.96
Mild liver disease	1	4.08	2.50 (1.69, 3.70)	2	52.60
Diabetes with complications	2	4.08	0.63 (0.42, 0.96)		29.22
Moderate or severe liver diseases	3	2.86	3.85 (2.41, 6.14)	4	62.96
Dementia	1	2.38	0.70 (0.43, 1.14)		33.33
Peptic ulcer disease	1	2.17	0.87 (0.51, 1.48)		31.70
Connective tissue-rheumatic diseases	1	1.85	0.91(0.52, 1.60)		31.43
HIV	6	0.11	1.26(0.11, 14.2)	1	25.00

† ICU weights calculated from logistic regression model. A blank space means that a null value was assigned to this co-morbidity.

**Figure 1:**
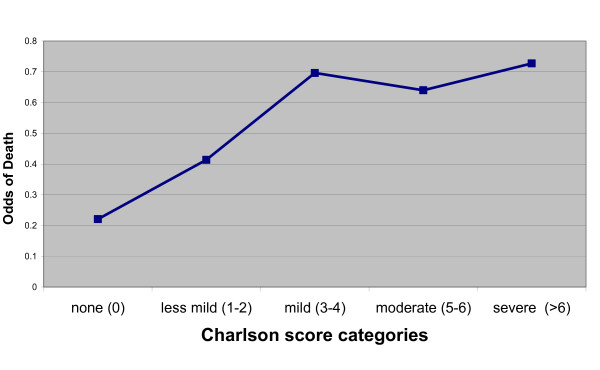
**The relationship between the odds of death for the Charlson score and categories was linear for index scores of 4 or less, but this relationship was no longer consistent for scores above 4**.

Table 3 displays the models constructed to predict hospital mortality. The CHP component of APACHE II alone was not a good univariate predictor of hospital mortality (C = 0.594). The Charlson index was a slightly better predictor (C = 0.626). In contrast, the full APACHE II was a good predictor of hospital mortality (C = 0.808). No further gain in discrimination was detected when the Charlson index was added to the full APACHE II model (C = 0.808 to C = 0.8135). In the nested multivariable models, the baseline model of age, sex and APS (Model A) performed reasonably well (C = 0.743). In each of the 3 subsequent nested models (Models B, C, or D) which added a measure of chronic health status to the baseline model, a statistically significant improvement in the model's discriminative ability to predict hospital death was observed (Model B, C = 0.757, Model C, C = 0.768, Model D, C = 0.752, p < 0.0001 each Model relative to Model A). However, the increase in discriminative ability would be considered of minimal practical significance. The Charlson index scores above 6 did not demonstrate a linear relationship with the log odds of death (Figure 1). Therefore, a post-hoc modification was performed where individual subjects with a Charlson index score of 6 and above were excluded (Model E). This analysis also demonstrated a statistical (C = 0.757) but not practical improvement in the discriminative ability to predict hospital death compared to the baseline model. Model B (assessment of the contribution of individual co-morbidities) was the most complex model with the largest AIC of 100.6. In contrast, when the Charlson index weighted scores were included (Models C and D), there was only a moderate increase (AIC = 38.90, AIC = 9.92 respectively).

**Table 3:** Model Performance for Predicting In-hospital Mortality

**Model**	**Log Likelihood**	**Degrees of Freedom**	**P value for LR**	**Likelihood Ratio Test**	**C stat**	**AIC** **(LR-2xdf)**	**Mean Shrinkage**
**Model A (Baseline model)** age sex APS *	-1882.01				0.743		0.97

**Model B** age sex APS CHP^†^	-1843.92	1	<0.0001	76.17	0.757	74.17	0.97
**Model C** Age, sex, APS, c. cob^‡^	-1814.69	17	<0.0001	134.64	0.768	100.64	0.94
**Model D (D'Hoore)** Age, sex, APS, Charlson score	-1861.56	1	<0.0001	40.89	0.752	38.89	0.98
**Model E** Age, sex, APS, Charlson score (excluded scores 6 and above)	-1701.14	1	<0.0001	11.92	0.757	9.92	1.01
**Model F** APACHE II, Charlson score	-1652.67				0.813		0.99
**Model G** APACHE II (with diagnosis)	-1670.11				0.808		1.00

*APS is the acute physiology score derived from APACHE II by subtracting the age and the CHP components

^† ^CHP is the Chronic Health Points from the APACHE II score.

‡Charlson co-morbidities entered as individual dummy variables

**Figure 2:**
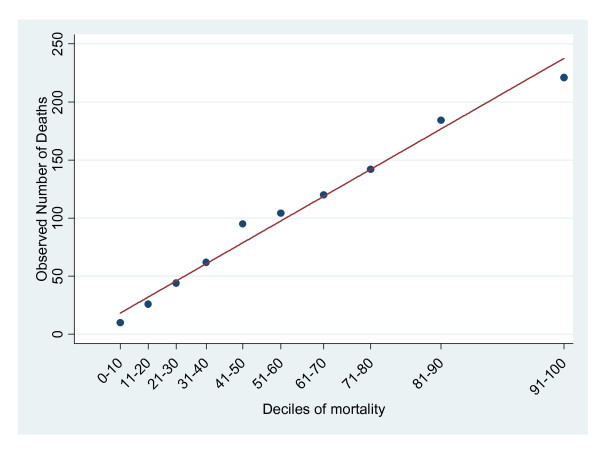
**This graph is a plot of observed and expected risk groups for each decile of in-hospital mortality**. Observed points falling on the line show good calibration for Model D.  Points falling above the line show that the model underestimated the actual risk of death.

Figure 2 shows a scatter plot of the expected vs. observed number of deaths for the Charlson index using the D'Hoore cut points (Model D). Data points falling on the 45 degrees line indicate perfect calibration. Most of the data points fell on or close to the line indicating fairly good calibration; both Models B and C had similar graphs (not shown).

## Discussion

The objective of this study was to assess the performance of the Charlson index alone and in combination with components of the APACHE II for predicting hospital mortality among adult ICU patients. Our results show as expected that the full APACHE II model performed the best of all models in predicting hospital mortality. The discrimination of the APACHE II model in our data (C = 0.808) was similar to the results from other large studies of similar duration and size [22], and similar to the results of other specific ICU acuity of illness taxonomies such as SAPS or MPM. A baseline model of age, sex, and APS provided good discriminative ability to predict mortality (C = 0.743). The addition of the Charlson co-morbidity components added little to either the baseline model, or to the full APACHE II model. Further, these results demonstrate and it is reasonable to conclude that adding or replacing the APACHE II CHP with the Charlson index provides no practical improvement to the discriminative ability of such a predictive model (C = 0.808 vs C = 0.813). Our results are similar to those published by Ho *et al. *These investigators from Royal Perth Hospital (which provides ICU care to a large region of Western Australia) demonstrated that more comprehensive measures of co-morbidity did not significantly improve the discrimination of the APACHE II score [23].

It may not be surprising that the Charlson index did not perform as well as APACHE II. APACHE II was originally developed and validated in ICU patients, whereas the Charlson index was developed using 1 year survival data on medical in-patients, with subsequent analysis assessing its ability to predict survival over 10 years for breast cancer patients. Since the population groups and outcomes are different, the weights applied in the Charlson index may not be appropriate for critically ill patients. For example, our data demonstrated that only 10/17 Charlson co-morbidities were associated with an increased risk of hospital death. Myocardial infarction and peripheral vascular disease were the only 2 co-morbidities that had the same original Charlson weights. Co-morbidities with an infrequent prevalence (less than 3%) such as dementia, connective tissue-rheumatic disease, and peptic ulcer disease were not associated with mortality, while HIV was assigned a much smaller weight compared to the original Charlson index. It may be that some of the co-morbidities, such as dementia, were associated with a clinical decision to exclude ICU admission, but our data can not ascertain if this form of selection bias was present, or if present, its potential magnitude. Another alternate explanation is that since the Charlson index was derived in 1987, advances in the treatment and therefore risk of mortality for some co-morbidities, for instance HIV/AIDS, has changed. Therefore, the current weights applied in the Charlson index may not be appropriate.

This study showed the Charlson index had a non-linear relationship with the odds of mortality, results similar to a study in ischemic heart patients [17]. The distribution of Charlson index scores in our results was skewed, with 77.23% of patients assigned a score of two or less. The small number of ICU patients in our sample with high Charlson index scores may explain why the estimate was unstable in this subset. Our results differ from those of Poses *et al.*, who demonstrated that the Charlson index, as a univariate predictor, had a linear relationship with hospital mortality for ICU patients [11]. The disparity in results may be due to their small sample size, (N = 201), or differences in the methods of calculating the Charlson Index as their results report a labor intensive chart review rather than the use of administrative data [11].

The present study had several strengths. All critically ill patients admitted to an ICU in the CHR during the study period were included in the sample, therefore reducing susceptibility to selection bias from referral, but not necessarily excluding bias in cases where critically ill patients were refused admission to ICU based on underlying co-morbidities and probability for death. Unfortunately, we do not have a method of determining if this occurred. Over-fitting of the models was reduced because we excluded complications using the diagnosis type indicator in the administrative data and this was assessed by bootstrapping methodology. Furthermore, we had a large sample size (N = 3778) and sufficient hospital mortality (27.1%), which produced stable estimates of coefficients that were less susceptible to shrinkage. Our study suggests that ICU specific weights may be quite different than the original Charlson weights, and these results should serve as caution to health service researchers when using the Charlson index as risk adjustment measure in ICU patients.

Although the Charlson index alone did not perform as well (C = 0.626) as the full APACHE II, we do not believe our results preclude its use for risk adjustment in administrative data in the absence of ICU acuity of illness scores. The collection of ICU acuity of illness scores is labour intensive and therefore costly. The validity of collecting APACHE II data from retrospective chart review has not, to our knowledge, been validated (for example, accurate assessment of Glasgow coma score may be very difficult). In contrast, the Charlson index is well suited for use in administrative datasets, and algorithms developed to recode administrative collected and coded diagnosis data into a Charlson index has been well studied and validated [8,9]. Therefore, the Charlson index may have practical applications when APACHE II or other acuity scores have not been prospectively determined, or have been determined in a non-standard manner. Furthermore, age and sex are data nearly always available in administrative datasets. When we analyzed the Charlson index with age and sex, the C-statistic increased to 0.68 (analysis not presented), supporting the utility of the Charlson index as a risk adjustment method in the absence of ICU based acuity scores. The Charlson index may also have some usefulness in comparing outcomes between ICU patient populations where the same acuity of illness score has not been used. Finally, the utility of ICU acuity illness scores in the assessment of outcome beyond hospital discharge has not been well established, whereas, the Charlson index is an accepted method of risk adjustment in longer term outcome studies in non-ICU patients. Future studies should explore the Charlson index's ability to predict longer term outcomes in ICU and whether modification of the Charlson index could help improve the performance of this risk adjustment method in intensive care data.

## Conclusion

The Charlson index does not perform as well as the APACHE II in predicting hospital mortality in ICU patients. However, when acuity of illness scores are unavailable or are not recorded in a standard way, the Charlson index might be considered as an alternative method of risk adjustment and therefore facilitate comparisons between intensive care units.

## Key messages

• When comparing health care delivery between ICUs risk adjustment methods are required.

• A well validated method of calculating Charlson index scores from administrative data can be used to obtain Charlson scores for individual ICU patients without an expensive, labour intensive chart review.

• Although the Charlson index does not perform as well at predicting in-hospital mortality among ICU patients as the APACHE II, the Index could be used when acuity of illness scores are unavailable or are not recorded in a standard way.

• Future studies should compare the ability of the Charlson index and the APACHE system to predict longer term outcomes in ICU.

• In addition, recalculating and updating Charlson weights could help to improve performance of this risk adjustment method in intensive care data.

## Abbreviations

APACHE II: Acute physiology and chronic health evaluation version II; ICU: Intensive care unit; CHR: Calgary Health Region; SAPS: Simplified Acute Physiology Score; MPM: Mortality Prediction Model; APS: Acute Physiology Score; QS: Quantitative Sentinel; PHN: Provincial healthcare number; ICD: International Classification of Disease; LOS: Length of stay; LR: Likelihood ratio; AIC: Akaike's Information Criterion

## Competing interests

The authors declare that they have no competing interests.

## Authors' contributions

SQ was involved in the concept and design of the study, carried out the data analysis, participated in the interpretation of data, drafted the article and gave final approval to be published. DH was involved in the concept and design of the study, participated in the interpretation of data, drafted the article, critically revised the manuscript and gave final approval to be published. PF was involved in the concept and design of the study, participated in the interpretation of data, critically revised the manuscript and gave final approval to be published. AF was involved in acquisition of the data, participated in interpretation of data, critically revised the manuscript and gave final approval to be published. HQ was inovled in the concept and design of the study, participated in the acquisition and interpretation of data, critically revised the manuscript and gave final approval to be published. CD was involved in the concept and design of the study, participated in the acquisition and interpretation of data, critically revised the manuscript and gave final approval to be published.

## Pre-publication history

The pre-publication history for this paper can be accessed here:


